# Surface-Enhanced Raman Spectroscopy to Characterize Different Fractions of Extracellular Vesicles from Control and Prostate Cancer Patients

**DOI:** 10.3390/biomedicines9050580

**Published:** 2021-05-20

**Authors:** Eric Boateng Osei, Liliia Paniushkina, Konrad Wilhelm, Jürgen Popp, Irina Nazarenko, Christoph Krafft

**Affiliations:** 1 Leibniz Institute of Photonic Technology, Member of Research Alliance “Health Technologies“, Albert-Einstein-Straße 9, 07745 Jena, Germany; eboateng@chem.ubc.ca (E.B.O.); juergen.popp@leibniz-ipht.de (J.P.); 2 Institute of Physical Chemistry and Abbe School of Photonics, Friedrich Schiller University Jena, Helmholtzweg 4, 07743 Jena, Germany; 3 Medical Center University Freiburg, Institute for Infection Prevention and Hospital Epidemiology, Faculty of Medicine, University of Freiburg, Hugstetter Str. 55, 79106 Freiburg, Germany; liliia.paniushkina@uniklinik-freiburg.de (L.P.); irina.nazarenko@uniklinik-freiburg.de (I.N.); 4 Center for Surgery, Medical Center, Department of Urology, Faculty of Medicine, University of Freiburg, Hugstetter Str. 55, 79106 Freiburg, Germany; konrad.wilhelm@uniklinik-freiburg.de; 5 German Cancer Consortium, Partner Site Freiburg and German Cancer Research Center (DKFZ), Hugstetter Str. 55, 79106 Freiburg, Germany

**Keywords:** extracellular vesicles, Raman spectroscopy, surface enhanced Raman spectroscopy

## Abstract

Extracellular vesicles (EVs) are membrane-enclosed structures ranging in size from about 60 to 800 nm that are released by the cells into the extracellular space; they have attracted interest as easily available biomarkers for cancer diagnostics. In this study, EVs from plasma of control and prostate cancer patients were fractionated by differential centrifugation at 5000× *g*, 12,000× *g* and 120,000× *g*. The remaining supernatants were purified by ultrafiltration to produce EV-depleted free-circulating (fc) fractions. Spontaneous Raman and surface-enhanced Raman spectroscopy (SERS) at 785 nm excitation using silver nanoparticles (AgNPs) were employed as label-free techniques to collect fingerprint spectra and identify the fractions that best discriminate between control and cancer patients. SERS spectra from 10 µL droplets showed an enhanced Raman signature of EV-enriched fractions that were much more intense for cancer patients than controls. The Raman spectra of dehydrated pellets of EV-enriched fractions without AgNPs were dominated by spectral contributions of proteins and showed variations in S-S stretch, tryptophan and protein secondary structure bands between control and cancer fractions. We conclude that the AgNPs-mediated SERS effect strongly enhances Raman bands in EV-enriched fractions, and the fractions, EV12 and EV120 provide the best separation of cancer and control patients by Raman and SERS spectra.

## 1. Introduction

Extracellular vesicles (EVs) are membrane-enclosed structures ranging in size from approximately 60 to 800 nm that are released from the cells into the extracellular space. EVs are composed of lipids, proteins, nucleic acids, and are involved in intercellular communication by transporting these molecules between distant cells [1]. EVs can be subdivided by their origin into exosomes, microvesicles, and apoptotic bodies. However, other, yet undetermined types of EVs may exist [1]. While exosomes with a typical size range of 60 to 120 nm originate from multivesicular bodies of the endosomal compartment, microvesicles, which typically range in size from 150 to 800 nm, are released from cells by direct budding from the outer cell membrane. Apoptotic bodies are considered to be even more heterogeneous and may range in size from 400 to approximately 1500 nm [1,2]. EVs are distributed among various body fluids such as blood, urine, saliva, cerebrospinal fluid, breast milk and ascites, and can serve as a valuable source of diagnostic biomarkers for health and disease, as recently reviewed elsewhere [3,4,5,6]. However, due to their heterogeneity, EV analysis and consequently their application in diagnostics still remain challenging because the existing technologies developed for macro- and micro-objects need to be adapted for the analysis of heterogeneous nano-sized targets. Among these techniques, high-resolution flow cytometry, nanoparticle tracking analysis, and tunable resistive pulse sensing are regarded as mostly well characterized and accepted in the field [7]. Along with the adaptation and further development of the existing methods, alternative techniques, e.g., Raman spectroscopy appear to be promising for label-free EV analysis for diagnostic purposes [8].

Usage of the spontaneous Raman spectroscopy has revealed that the EV-specific signal is weak for the detection of single EVs and the sensitivity of the method is low for the characterization of highly heterogeneous EV populations. The Raman spectra of bulk EV samples provides average signatures of EVs with substantially higher signal intensities than those of a single EV [9,10]. However, the challenge of performing a single EV analysis was achieved by trapping single vesicles by optical tweezers followed by Raman spectroscopy [11,12,13,14,15]. As a promising alternative to spontaneous Raman, the surface enhanced Raman scattering (SERS) effect, which allows an increase in the sensitivity of measurements by a plasmonic interaction between analytes and nanostructured surfaces, was considered [16,17]. SERS approaches can be roughly divided into SERS probes and SERS substrates, while both can be performed with or without immuno-labeling. SERS-active nanoparticles for EV detection were prepared from gold [18,19] or silver [20]. To improve the specificity of SERS, nanorods and magnetic nanobeads were decorated with antibodies enabling the capture of EVs [21], or with aptamers as alternative biorecognition elements [22]. SERS-active substrates were introduced for EV analysis using super-hydrophobic surfaces in the form of patterned silicone micro-pillars decorated with silver nanoaggregates [23] or nanobowls coated with a silver film [24].

In this manuscript, we compare the performance of SERS (using silver nanoparticles, AgNPs) and spontaneous Raman spectroscopy in a proof-of-concept study aiming to identify tumor-characteristic signatures of EV-enriched fractions isolated from plasma of patients with prostate carcinoma (PCa). As a control, we used EVs isolated from the plasma of patients with benign hypoplasia (BPH). Our data indicate that SERS is advantageous due to the signal enhancement, which allows better discrimination between tumor- and non-tumor samples.

## 2. Materials and Methods

### 2.1. Patient Characteristics

Blood samples for this study were collected from patients undergoing prostate needle biopsy due to elevated PSA-values at the Department of Urology at the Medical Center, University of Freiburg. They had a confirmed clinical and histopathological diagnosis, i.e., either exclusion or diagnosis of PCa. The patients with PCa had a needle biopsy confirming high-risk prostate cancer (≥Gleason 7b) and the control group had tumor exclusion by needle biopsy. Patients were equal in age, BMI, prostate volume and PSA level (Supplementary Materials, Table S1). Patients with diabetes or known history of systematic inflammatory diseases were excluded from the sampling. Ethical approval to study the human blood citrate plasma samples was obtained by the Institutional Review Board of the Department for Urology (Freiburg, Germany). This study was conducted in accordance with the standards laid down in the 1964 Declaration of Helsinki and its later amendments. Informed consent was obtained from all participating individuals.

### 2.2. Preparation of EV Crude Fractions

**Figure 1:**
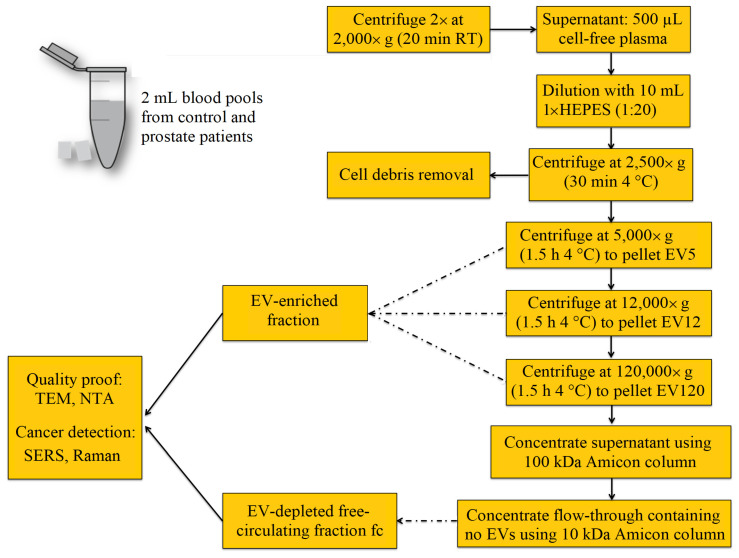
EV enrichment and fractionation protocol from blood using differential centrifugation resulted in three crude EV-enriched fractions designated as EV5, EV12 and EV120, and one EV-depleted free circulating fraction (fc).

The procedure for the isolation of crude fractions of blood-derived EVs is presented in Figure 1. Blood samples were collected using citrate plasma vacutainers. To remove remaining cells including platelets, the samples were centrifuged twice at 2000× *g* for 20 min at room temperature. Then, three pools were generated: (1) a control pool, for which four plasma samples of patients with benign hypoplasia were mixed together; and (2) pool A and pool B, both of which consisted of plasma from four patients with prostate cancer. Then, the cell-free plasma was divided into aliquots and stored at −80 °C until further processing. For isolation of crude EV fractions, the 500 μL aliquots were thawed briefly in a water bath and diluted with 10 mM HEPES in the ratio (1:20). The samples were centrifuged at 2500× *g* for 30 min, at 4 °C. To separate the EV5 fraction, the supernatants were centrifuged at 5000× *g* for 1.5 h, at 4 °C. The pellets (EV5) were collected and the supernatants were used to isolate the EV12 fractions by centrifugation at 12,000 *g* for 1.5 h, at 4 °C. Then, the EV120 fractions were isolated by filtration through a 0.2 µm filter followed by the ultracentrifugation at 120,000× *g* for 1.5 h, at 4 °C. The supernatant of EV120 was concentrated using ultrafiltration, with a cut-off by 100 kDa. The flow-through, considered as an EV-free circulating fraction (fc) was further concentrated using ultrafiltration with the 10 kDa cut-off. All samples were stored at −80 °C until further EV characterization and spectroscopic analysis.

### 2.3. Characterization of EV Fractions by DLS, NTA and TEM

The quality of all vesicle preparations was controlled according to the recommendations of the MISEV guidelines 2018, specifically, dynamic light scattering (DLS), nanoparticle tracking analysis (NTA), and transmission electron microscopy (TEM) were performed [4]. DLS was carried out using the Nano-flex 180 DLS system (Particle Metrix, Meerbusch, Germany), according to the manufacturer’s instructions. In brief, 20 μL of undiluted sample was placed on the measuring probe. Each sample was measured 5 times for 60 s and the results were averaged. Results were transferred from Intensity mode to Volume mode. The setup was as follows: Refractive Index 1.33; Min Size 0.8 nm; Max Size 6540 nm; Viscosity: Low Temperature 1.002; High Temperature 0.797; Transparency “on”; Shape: spherical. Estimation of the particles’ size using DLS was done with each of the fractions, including EV5, EV12, EV120 and fc. The particle concentration of these fractions was determined by NTA using the Zeta View system PMX110 (Particle Metrix, Meerbusch, Germany). According to the manufacturer’s protocol, samples were diluted with 10 mM HEPES 1:500 and one mL of diluted sample was injected into the NTA. The size distribution was measured in scatter mode and Zeta potential was measured in the Zeta Potential mode using the following setup: Sensitivity 85; Min size 20 nm; Max size 1000 nm; Min Brightness 10. Images were recorded at 11 positions and 5 cycles with a camera sensitivity of 65–81% and temperature monitored manually, ranging from 21 to 22 °C. As a negative control 10 mM HEPES solution was used. The particle number measured in the control was subtracted from the particle numbers measured in the samples. All measurements were performed in triplicate, and the mean value was calculated. The EV120 and fc fractions were additionally characterized by TEM. A 10 µL sample was loaded on a 300-mesh copper grid and fixed with 1% glutaraldehyde. Next, it was washed with double distilled water and negatively stained with 10 µL drop of 1% uranyl acetate. The images were taken using an electron microscope (LEO 906 E, Zeiss, Oberkochen, Germany) using SIS software (Olympus, Hamburg, Germany).

### 2.4. Preparation of SERS-Active Nanoparticles

The silver nanoparticle solution was synthesized by the reduction of silver nitrate (Sigma Aldrich, St. Louis, MO, USA) at room temperature using hydroxylamine hydrochloride according to the protocol described by Leopold and Lendl [25]. To enrich mostly monodispersed particles, 10 mL of 1 mM silver nitrate was added dropwise to 90 mL of 1.5 M hydroxylamine/sodium hydroxide containing 3 mM sodium hydroxide (Sigma Aldrich, St. Louis, MO, USA). The reaction was carried out at room temperature. While stirring the reaction mixture, a color change to yellow was observed with AgNPs with an average size of around 50 nm. To induce AgNP aggregation, equal volume of 1 M potassium chloride (Sigma Aldrich, St. Louis, MO, USA) was added immediately before Raman experiments. The concentration of AgNPs was calculated to be 0.1 mg/mL before aggregation and 0.05 mg/mL after adding an equal volume of potassium chloride.

### 2.5. Raman and SERS Measurement

The Raman and SERS spectra were collected with the Raman spectrometer RXN1 using 785 nm laser excitation coupled to the microscope (Kaiser Optical System, Ann Arbor, MI, USA). For SERS data acquisition, the AgNP suspension was mixed with an equal volume of potassium chloride on a super mirror stainless steel substrate [26] (Renishaw PLC, Wotton-under-Edge, UK), followed by adding the EV-enriched or fc fraction in a mass ratio of 20:1 (EV:AgNP) in a final volume of 10 µL. The laser with output power of 150 mW was focused on the droplet surface via a 10×/0.25 microscope objective lens (Leica Microsystems, Wetzlar, Germany). Five SERS spectra were acquired with 10 s acquisition time, and an average spectrum was calculated for each droplet. For Raman data acquisition, 5 μL of EV-enriched or fc fraction was deposited on a CaF_2_ substrate without AgNPs and allowed to dry. Raman images covering the margin of the dried sample film were collected with a laser power of 150 mW, 100×/0.90 objective lens (Nikon, Tokyo, Japan), 10 s exposure time and a step size of 10 μm. Photomicrographs of dried sample films were then taken with a digital microscope (Keyence VHX-500 with zoom objective VH-Z100UR, Osaka, Japan).

### 2.6. Data Analysis

The Raman images were imported to Holomap (Kaiser) which runs under Matlab (Mathworks, Portola Valley, CA, USA) to select the most intense spectra for averaging. Further spectral processing such as baseline correction, smoothing and intensity normalization was performed with the hyperSpec package [27] within the R software environment [28]. For comparison, the Raman spectra were normalized for equal intensities of the protein amide I band near 1660 cm^−1^. Difference spectra were calculated between control and cancer fractions. SERS spectra were only corrected for baseline and not normalized for comparison of signal enhancements.

## 3. Results and Discussion

### 3.1. Characterization of EV and fc Fractions by SERS

**Figure 2:**
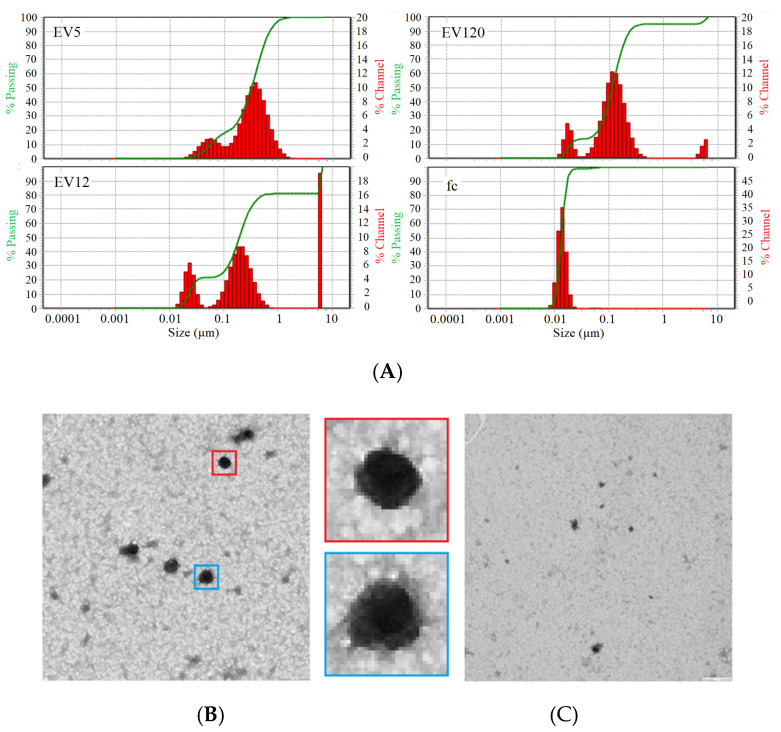
(**A**) Particle size distribution by dynamic light scattering of EV5, EV12, EV120 and fc fractions isolated from pools of citrate plasma samples. (**B**) Transmission electron microscopy of EV120-enriched fraction, two EVs at five-fold enlargement (blue and red box) showing typical EV-like structures of approximately 100 nm diameter and (C) fc fraction.

To ensure the comparability and reproducibility of SERS measurements among different fractions, the AgNP with EVs were mixed in a constant ratio. For that, data acquired by DLS (Figure 2A), NTA (Table 1) and TEM (Figure 2B,C) were used to approximate the mass concentration of particles. With the help of DLS data, diameter of particles in each of the fractions was assessed, and assuming that EVs are spherical, the volume was calculated as follows:

(1) $$V=4/3\;\pi \cdot r^{3}$$

Presuming particle density of 1 mg/mL, mass concentration was calculated as a function of volume, particle number/mL acquired by NTA, and mass (Table 1). This concentration was used to calculate constant mass ratios of 20 to 1 of EVs and AgNPs.

### 3.2. SERS Spectra of EV-Enriched and fc Fractions

**Figure 3:**
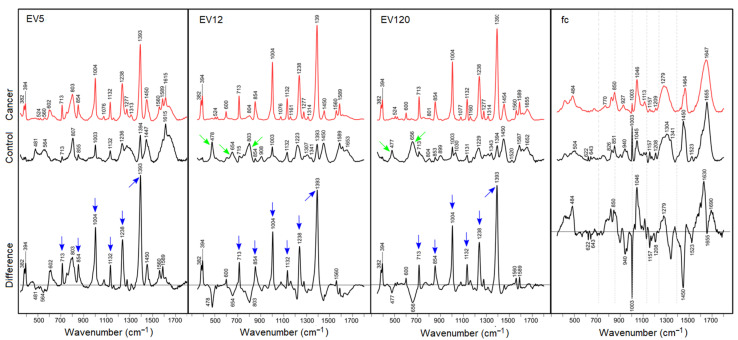
SERS spectra at 785 nm excitation mixing AgNPs, potassium chloride and plasma fractions EV5, EV12, EV120 and fc. Blue arrows denote most intense SERS bands of EV-enriched fractions in prostate cancer samples, green arrows denote more intense SERS bands of proteins in control samples.

The SERS spectra from crude preparations of EVs generated by differential centrifugation and designated as EV5, EV12, and EV120 according to the centrifugation speed, and the fc fraction of control and cancer samples are shown in Figure 3. Despite the low EV concentration, mixing the fractions with small volumes of AgNPs and potassium chloride to achieve mass ratios of 20 to 1 of EVs and AgNPs significantly enhanced the Raman signal intensities.

SERS bands of cancer-derived EV-enriched fractions were well resolved near 382, 394, 600, 713, 854, 1004, 1132, 1238, 1393, 1450, 1560 and 1589 cm^−1^. SERS bands of control EV-enriched fractions were remarkably different, in particular intensities relative to the band near 1450 cm^−1^, which shows only small intensity variations. SERS bands of control EV fractions were found near 478, 656, 803, 899, 1003, 1030, 1132, 1223, 1343, 1393, 1450, 1589, 1615 and 1653 cm^−1^. The underlined SERS bands, which show weaker counterparts in SERS spectra of cancer EV fractions, were previously detected in SERS spectra of cell lysates near 660, 800, 895, 1028, 1218, 1339, 1450, 1588 and 1660 cm^−1^ [29]. Difference spectra were calculated to visualize the most significant changes between cancer and control fractions. Positive difference bands were consistently observed near 382, 394, 600, 713, 854, 1004, 1132, 1238, 1393, 1560 and 1589 cm^−1^, and negative difference bands near 477 and 656 cm^−1^, whereas the band ratios 1393 cm^−1^ to 1450 cm^−1^ were close to one for all control EV fractions, and the ratio was maximum for EV12, followed by EV120 and EV5 of cancer fractions. As the intensity scales for the spectra in Figure 3 were not identical, the positive difference bands were integrated for quantitation. The intensities were 153,163 for EV5, 672,261 for EV12 and 290,069 for EV120 which demonstrates that the series of SERS bands were maximum for EV12. All difference spectra were overlaid at the same scale for visualization (Supplementary Material, Figure S1). SERS enhancement was generally weak in both fc fractions, and the potential markers for cancer-derived EV-enriched fractions were absent. Instead, well-known Raman bands of proteins at 643, 826, and 850 cm^−1^ due to tyrosine, at 622, 1003, and 1209 cm^−1^ due to phenylalanine, at 940, 1300 and 1655 cm^−1^ due to the peptide backbone, and at 1340 and 1450 cm^−1^ due to aliphatic aromatic acids were identified. More variations were evident in the difference spectrum between cancer and control fc fractions. Positive bands near 484, 1046, 1629 and 1690 cm^−1^ were tentatively assigned to buffer, and negative difference bands to proteins as described above, and at 1157 and 1523 cm^−1^ to carotenoids. Small standard deviations around mean SERS spectra of control and cancer EV-enriched fractions indicate the high reproducibility of all band intensities, in particular, the series of strongly enhanced bands in cancer-derived EV-enriched fractions (Supplementary Materials, Figure S2). Based on these data, we cannot conclude yet which of the components of the preparation provided these SERS signals, as the crude EV preparations, containing a high portion of lipoproteins and large protein aggregates were used in this initial study. However, these data point towards the strong advantages of the AgNP-supported SERS, opening a new avenue for SERS-based characterization of EV populations. Our next efforts will focus on the characterization of different, highly pure EV populations, lipoproteins and other blood plasma components in cancer versus non-cancer patients in order to determine the most promising blood compartments, allowing the best separation of cancer and cancer-free samples.

### 3.3. Raman Spectra of EV-Enriched and fc Fractions

**Figure 4:**
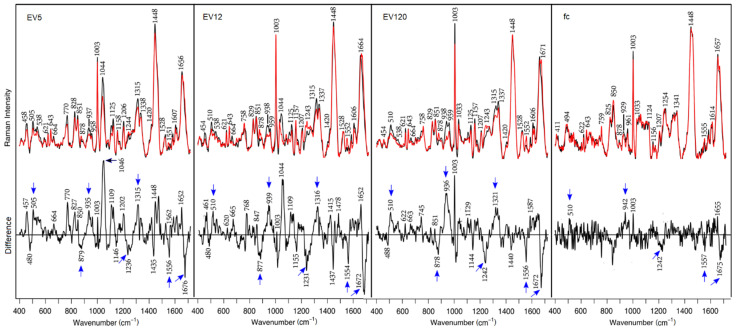
Raman spectra at 785 nm excitation from dried serum fractions EV5, EV12, EV120 and fc. Control (black trace), cancer (red trace). Reproducible difference features are labeled by blue arrows.

Raman spectra of control and cancer EV-enriched and fc fractions are overlaid for comparison in Figure 4. Difference spectra between control and cancer samples are included. Similar data have previously been presented for EV12 and EV120, but not for EV5 and fc fractions. Therefore, the new data demonstrate the reproducibility and extend the previous findings. All spectra are dominated by protein bands with only small variations between control and cancer fractions. The Raman spectra and—even more importantly—the difference spectra share many similarities with previously reported data [10]. Main bands are assigned to disulfide bonds (505 and 538 cm^−1^), aromatic amino acids Phe (621, 1003, 1207 and 1606 cm^−1^), Tyr (643, 828 and 851 cm^−1^), Trp (758, 878 and 1552 cm^−1^), aliphatic amino acids (1125, 1337 and 1448 cm^−1^), the peptide groups (937, 1243, 1315 and 1660 cm^−1^) and carotenoids (1157 and 1528 cm^−1^). Bands near 458, 770 and 1044 cm^−1^, which are maximum in fraction EV5 and decline in fractions EV12 and EV120, cannot be assigned yet. Difference spectra were calculated to visualize changes between control and cancer fractions. Reproducible positive difference bands were observed near 510, 935, 1315 and 1652 cm^−1^, and reproducible negative bands were seen near 879, 1242, 1554 and 1672 cm^−1^. These features point to elevated content of proteins with α-helical secondary structures (935, 1315 and 1652 cm^−1^) and disulfide bonds (510 cm^−1^) in control fractions, and elevated content of proteins with β-sheet secondary structures (1242 and 1672 cm^−1^) and changes in tryptophan amino acids (878 and 1555 cm^−1^) in cancer fractions. Similar to the SERS difference spectra, these difference bands were integrated after normalization to the band at 1448 cm^−1^. The absolute intensities were 7205 for EV5, 13,908 for EV12 and 17,251 for EV120. Here, the difference intensities were most intense for EV120, followed by EV12. The Raman spectra of fc fractions show the lowest intensities. Remarkable changes in fc fractions relative to the other fractions are the different band ratios of the Tyr doublet at 825 and 850 cm^−1^, the amide III band maximum at 1254 cm^−1^, and the absence of detectable carotene amounts (no band near 1530 cm^−1^). The change in the amide III band points to the deviated protein composition of fc fractions with less ordered structures, which is typical for low molecular weight proteins. In spite of the weak differences, the tendency of the main positive and negative difference features is still evident in fractions fc. The standard deviations around the mean Raman spectra of control and cancer fractions (Supplementary Materials, Figure S3) tend to be larger for the less concentrated fc fractions and for the cancer-derived EV fractions due to more concentration variations according to Table 1.

Dried films of EV-enriched and fc fractions were also measured by AgNP-supported SERS. However, no usable Raman spectra were collected from these samples as no bands were resolved, no enhancing effects were evident, and the spectra were dominated by intense background signals. Representative microscopic images of each fraction are presented in the Supplementary Materials, Figure S4.

## 4. Conclusions

A protocol was described for preparing different fractions from plasma, mixing them with AgNPs and collecting high quality SERS spectra from these suspensions. Raman bands were enhanced for fractions with enriched EVs content, but not for EV-depleted fc fractions. In particular, a series of bands at 382, 394, 713, 854, 1004, 1132, 1238 and 1393 cm^−1^ showed high enhancement for cancer samples, which can be considered as potential cancer biomarkers. Maximum intensities in the fraction EV12 and less intense bands in EV120 and EV5 suggest that these crude fractions contain some components that provide these signals. A very important next step will be the identification of these components, e.g., certain EV populations that provide these signals, as our current state of knowledge is insufficient. However, our results allow the exclusion of the EV-depleted fc fractions as candidates for Raman-based analysis, as these fractions did not exhibit a clear cancer versus non-cancer Raman signature. Other issues that need to be investigated further are sensitivity, i.e., what is the limit of detection for cancer-derived EVs, and specificity, i.e., whether the set of SERS bands is similar or different for other cancer-derived EVs.

Raman spectra were also collected from dried pellets without AgNPs. The previously found spectral features near 939, 1243, 1315 and 1671 cm^−1^, which distinguish control and cancer samples were best resolved in EV-enriched fractions EV12 and EV120. More features were identified that were assigned to disulfide bonds and tryptophan residues in proteins.

Although these first data suggest the advantage of SERS compared to Raman spectroscopy and established a correlation between SERS and Raman markers with prostate cancer, sample fractionation is very critical. Lipoproteins or protein aggregates may co-purify with EVs. This will be studied in future investigations aiming at precise assignment of the identified signals to certain blood compartments such as the highly purified EV population, lipoproteins, exomeres or other.

## Figures and Tables

**Table 1:** Results of NTA and DLS analysis, calculated concentrations of EV-enriched and fc fractions from control and cancer patient samples designated as Pool A and Pool B.

	Particle Number from NTA (10^9^/mL)			Approximated Diameter (nm)	Approximated Concentration (mg/mL)		
Sample	Control	Pool A	Pool B		Control	Pool A	Pool B
EV5	414	978	3029	500	27	63	197
EV12	1494	3206	16,332	200	6.3	13	69
EV120	960	2014	2817	100	0.5	1.0	1.4
fc	1116	2235	662	<50	0.07	0.15	0.04

## Data Availability

Data are available at the GitHub repository under https://github.com/e-boateng/Raman-SERS-characterization-of-EVs (accessed on 12 March 2021).

## Supplementary Materials

The following are available online at https://www.mdpi.com/article/10.3390/biomedicines9050580/s1, Table S1: Patients’ clinical characteristics. Figure S1: Overlay of SERS difference spectra, Figure S2: Standard deviations around mean SERS spectra, Figure S3: standard deviation around Raman spectra of control and cancer EV-enriched fractions, Figure S4: Microscopic images of EV-enriched and fc control fractions subjected to drop-coating deposition and dried on a CaF_2_ substrate.

## References

[1] Yanez-Mo M., Siljander P.R., Andreu Z., Zavec A.B., Borras F.E., Buzas E.I., Buzas K., Casal E., Cappello F., Carvalho J. (2015). Biological properties of extracellular vesicles and their physiological functions. J. Extracell. Vesicles.

[2] Kim D.K., Lee J., Kim S.R., Choi D.S., Yoon Y.J., Kim J.H., Go G., Nhung D., Hong K., Jang S.C. (2015). EVpedia: A community web portal for extracellular vesicles research. Bioinformatics.

[3] Fais S., O’Driscoll L., Borras F.E., Buzas E., Camussi G., Cappello F., Carvalho J., Cordeiro da Silva A., Del Portillo H., El Andaloussi S. (2016). Evidence-Based Clinical Use of Nanoscale Extracellular Vesicles in Nanomedicine. ACS Nano.

[4] Thery C., Witwer K.W., Aikawa E., Alcaraz M.J., Anderson J.D., Andriantsitohaina R., Antoniou A., Arab T., Archer F., Atkin-Smith G.K. (2018). Minimal information for studies of extracellular vesicles 2018 (MISEV2018): A position statement of the International Society for Extracellular Vesicles and update of the MISEV2014 guidelines. J. Extracell. Vesicles.

[5] O’Driscoll L., Stoorvogel W., Thery C., Buzas E., Nazarenko I., Siljander P., Yanez-Mo M., Fais S., Giebel B., Yliperttula M. (2017). European Network on Microvesicles and Exosomes in Health and Disease (ME-HaD). Eur. J. Pharm. Sci. Off. J. Eur. Federation Pharm. Sci..

[6] Słomka A., Mocan T., Wang B., Nenu I., Urban S.K., Gonzalez-Carmona M.A., Schmidt-Wolf I.G.H., Lukacs-Kornek V., Strassburg C.P., Spârchez Z. (2020). EVs as Potential New Therapeutic Tool/Target in Gastrointestinal Cancer and HCC. Cancers.

[7] Welsh J.A., Van Der Pol E., Arkesteijn G.J.A., Bremer M., Brisson A., Coumans F., Dignat-George F., Duggan E., Ghiran I., Giebel B. (2020). MIFlowCyt-EV: A framework for standardized reporting of extracellular vesicle flow cytometry experiments. J. Extracell. Vesicles.

[8] Nazarenko I. (2020). Extracellular Vesicles: Recent Developments in Technology and Perspectives for Cancer Liquid Biopsy. Recent Results Cancer Res..

[9] Gualerzi A., Niada S., Giannasi C., Picciolini S., Morasso C., Vanna R., Rossella V., Masserini M., Bedoni M., Ciceri F. (2017). Raman spectroscopy uncovers biochemical tissue-related features of extracellular vesicles from mesenchymal stromal cells. Sci. Rep..

[10] Krafft C., Wilhelm K., Eremin A., Nestel S., von Bubnoff N., Schultze-Seemann W., Popp J., Nazarenko I. (2017). A specific spectral signature of serum and plasma-derived extracellular vesicles for cancer screening. Nanomedicine Nanotechnol. Biol. Med..

[11] Carney R.P., Hazari S., Colquhoun M., Tran D., Hwang B., Mulligan M.S., Bryers J.D., Girda E., Leiserowitz G.S., Smith Z.J. (2017). Multispectral optical tweezers for biochemical fingerprinting of CD9-positive exosome subpopulations. Anal. Chem..

[12] Kruglik S.G., Royo F., Guigner J.-M., Palomo L., Seksek O., Turpin P.-Y., Tatischeff I., Falcón-Pérez J.M. (2019). Raman tweezers microspectroscopy of circa 100 nm extracellular vesicles. Nanoscale.

[13] Lee W., Nanou A., Rikkert L., Coumans F.A., Otto C., Terstappen L.W., Offerhaus H.L. (2018). Label-Free Prostate Cancer Detection by Characterization of Extracellular Vesicles Using Raman Spectroscopy. Anal. Chem..

[14] Smith Z.J., Lee C., Rojalin T., Carney R.P., Hazari S., Knudson A., Lam K., Saari H., Ibañez E.L., Viitala T. (2015). Single exosome study reveals subpopulations distributed among cell lines with variability related to membrane content. J. Extracell. Vesicles.

[15] Tatischeff I., Larquet E., Falcón-Pérez J.M., Turpin P.-Y., Kruglik S.G. (2012). Fast characterisation of cell-derived extracellular vesicles by nanoparticles tracking analysis, cryo-electron microscopy, and Raman tweezers microspectroscopy. J. Extracell. Vesicles.

[16] Rojalin T., Phong B., Koster H.J., Carney R.P. (2019). Nanoplasmonic Approaches for Sensitive Detection and Molecular Characterization of Extracellular Vesicles. Front. Chem..

[17] Cialla-May D., Zheng X.S., Weber K., Popp J. (2017). Recent progress in surface-enhanced Raman spectroscopy for biological and biomedical applications: From cells to clinics. Chem. Soc. Rev..

[18] Park J., Hwang M., Choi B., Jeong H., Jung J.-h., Kim H.K., Hong S., Park J.-h., Choi Y. (2017). Exosome Classification by Pattern Analysis of Surface-Enhanced Raman Spectroscopy Data for Lung Cancer Diagnosis. Anal. Chem..

[19] Stremersch S., Marro M., Pinchasik B.E., Baatsen P., Hendrix A., De Smedt S.C., Loza-Alvarez P., Skirtach A.G., Raemdonck K., Braeckmans K. (2016). Identification of Individual Exosome-Like Vesicles by Surface Enhanced Raman Spectroscopy. Small.

[20] Lee C., Carney R., Lam K., Chan J.W. (2017). SERS analysis of selectively captured exosomes using an integrin-specific peptide ligand. J. Raman Spectrosc..

[21] Zong S., Wang L., Chen C., Lu J., Zhu D., Zhang Y., Wang Z., Cui Y. (2016). Facile detection of tumor-derived exosomes using magnetic nanobeads and SERS nanoprobes. Anal. Methods.

[22] Wang Z., Zong S., Wang Y., Li N., Li L., Lu J., Wang Z., Chen B., Cui Y. (2018). Screening and multiple detection of cancer exosomes using an SERS-based method. Nanoscale.

[23] Tirinato L., Gentile F., Di Mascolo D., Coluccio M.L., Das G., Liberale C., Pullano S.A., Perozziello G., Francardi M., Accardo A. (2012). SERS analysis on exosomes using super-hydrophobic surfaces. Microelectron. Eng..

[24] Lee C., Carney R.P., Hazari S., Smith Z.J., Knudson A., Robertson C.S., Lam K.S., Wachsmann-Hogiu S. (2015). 3D plasmonic nanobowl platform for the study of exosomes in solution. Nanoscale.

[25] Leopold N., Lendl B. (2003). A new method for fast preparation of highly surface-enhanced Raman scattering (SERS) active silver colloids at room temperature by reduction of silver nitrate with hydroxylamine hydrochloride. J. Phys. Chem. B.

[26] Lewis A.T., Gaifulina R., Isabelle M., Dorney J., Woods M.L., Lloyd G.R., Lau K., Rodriguez-Justo M., Kendall C., Stone N. (2017). Mirrored stainless steel substrate provides improved signal for Raman spectroscopy of tissue and cells. J. Raman Spectrosc..

[27] Beleites C., Sergo V. Hyperspec: A Package to Handle Hyperspectral Data Sets in R. http://hyperspec.r-forge.r-project.org.

[28] CoreTeam R. (2012). R: A Language and Environment for Statistical Computing.

[29] Hassoun M., Köse N., Kiselev R., Kirchberger-Tolstik T., Schie I., Krafft C., Popp J. (2018). Quantitation of acute monocytic leukemia cells spiked in control monocytes using surface-enhanced Raman spectroscopy. Anal. Methods.

