# Supplementary material for: Surface-Enhanced Raman Spectroscopy to Characterize Different Fractions of Extracellular Vesicles from Control and Prostate Cancer Patients
Source: Biomedicines. 2021 May 20;9(5):580. doi: 10.3390/biomedicines9050580 (PMC8161280; doi:10.3390/biomedicines9050580)
Supplement: Supplementary file 1 [file biomedicines-09-00580-s001.zip › biomedicines-1158805-SI.pdf]

biomedicines

MDPI

Supplementary Information

Surface-enhanced Raman spectroscopy to characterize different fractions of extracellular vesicles from control and prostate cancer patients

Eric Boateng Osei [1,2,†], Liliia Paniushkina [3], Konrad Wilhelm [4], Jürgen Popp [1,2], Irina Nazarenko [3,5] and Christoph Krafft [1,*]

1 Leibniz Institute of Photonic Technology; Member of Research Alliance "Health Technologies", Albert-Einstein-Straße 9, 07745 Jena, Germany; eboateng@chem.ubc.ca (E.B.O.).
2 Institute of Physical Chemistry and Abbe School of Photonics; Friedrich Schiller University Jena, Helmholtzweg 4, 07743 Jena, Germany; juergen.popp@leibniz-ipht.de (J.P.)
3 Medical Center University Freiburg, Institute for Infection Prevention and Hospital Epidemiology, Faculty of Medicine, Freiburg, Germany. Hugstetter Str. 55, 79106 Freiburg, Germany, liliia.paniushkina@uniklinik-freiburg.de (L.P.) Irina.nazarenko@uniklinik-freiburg.de (I.N.)
4 Center for Surgery, Medical Center, Department of Urology, Faculty of Medicine, University of Freiburg, Hugstetter Str. 55, 79106 Freiburg, Germany konrad.wilhelm@uniklinik-freiburg.de (K.W.)
5 German Cancer Consortium (DKTK), Partner Site Freiburg and German Cancer Research Center (DKFZ), Heidelberg, Germany
* Correspondence: Christoph.krafft@leibniz-ipht.de; Tel.: +49 3641 206306
† Current address: Department of Chemistry, The University of British Columbia, 2036 Main Mall, Vancouver, BC V6T 1Z1, Canada

Table S1 Patients clinical characteristics

Patient diagnosis	N	Age (y)	BMI (kg/m²)	Prostate volume	PSA (ng/ml)	Gleason Score
**BPH**	1	58	36.3	35	4.97	n.a.
	2	60	31.1	65	8.2	n.a.
	3	70	23.4	40	2.64	n.a.
	4	71	23.9	30	2.9	n.a.
**Prostate cancer**	1	69	42.6	48	6.86	7b
	2	62	32	50	4.9	8
	3	63	22	40	6.71	9
	4	67	21	25	5.46	7b
**p-value (mean BPH vs. prostate cancer)**		0.37	0.22	0.93	0.73	n.a.

n.a (not applicable)

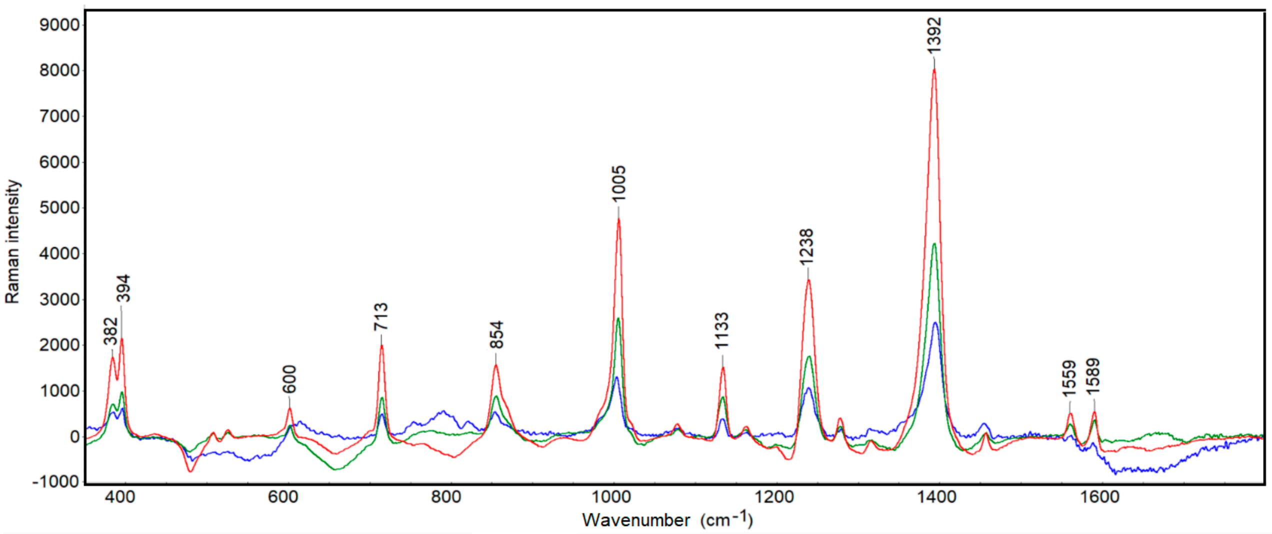

SI Figure 1: Overlay of SERS difference spectra for EV5 (blue), EV12 (red) and EV120 (green) shown on the same intensity scale. Highest intensities were obtained for EV12 followed by EV120 and EV5.

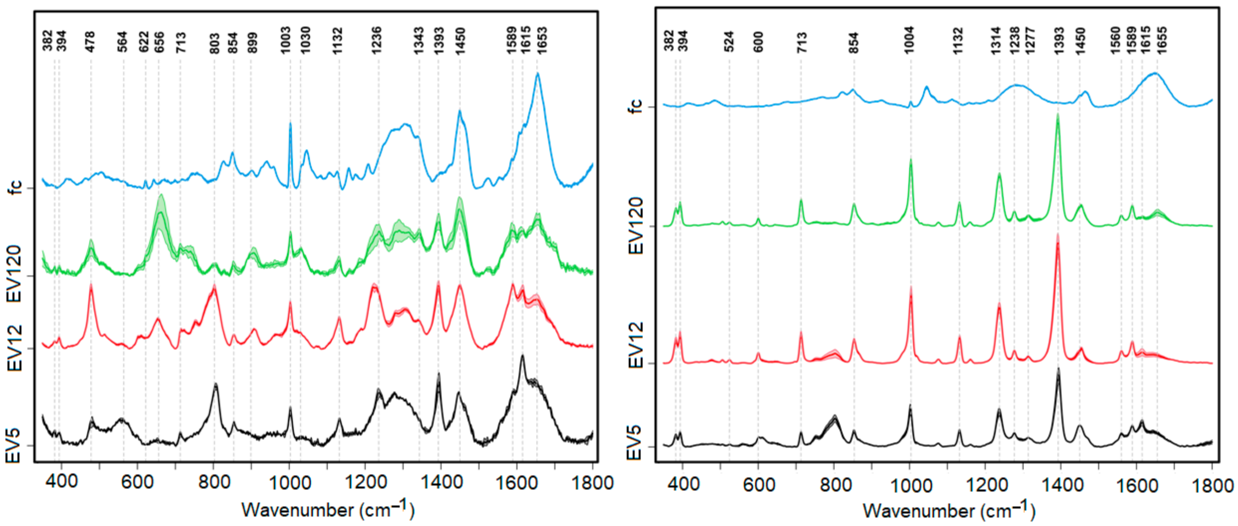

SI Figure 2: The mean SERS spectra ± standard deviation of control (left) and cancer (right) fractions. SERS bands (382, 394, 713, 854, 1004, 1132, 1238, 1393, 1560 and 1589 cm^{-1}) were integrated for quantitation.

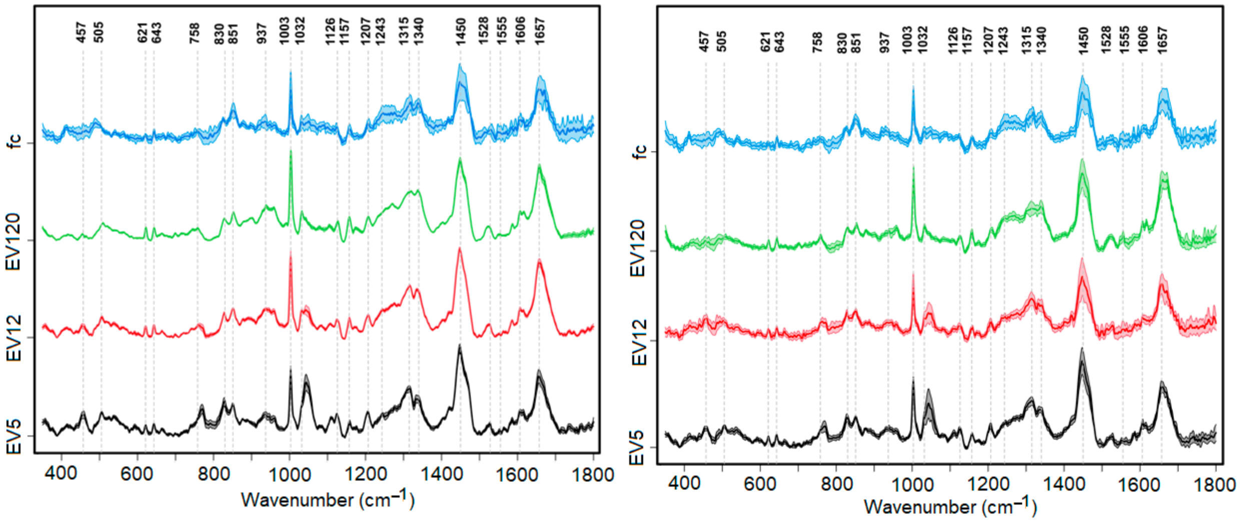

SI Figure 3: The mean Raman spectra ± standard deviation of control (left) and cancer (right) fractions. The Raman spectra of dried EVs without silver nanoparticles are dominated by spectral contributions of proteins.

Dried films of control and cancer EV samples were prepared for Raman spectroscopy by the drop coating deposition procedure. SI Figure 1 shows microscopic images of control EV5-, EV12-, EV120-, and fc-enriched fractions after drying on the calcium fluoride slide. The dried films formed a "coffee ring" with the highest sample concentration near the margin. This principle is based on the sessile drop formation [1]. Whereas the films of EV5, EV12 and EV120 looked similar, the ring of the fc fraction was very thin. Clusters of particles were evident at the center of the fc-AgNP film that were assigned to AgNPs and KCl crystals. Raman spectra were collected from each sample along the "coffee ring" as previously described [2]. However, no usable Raman spectra were collected from FC-AgNP films.

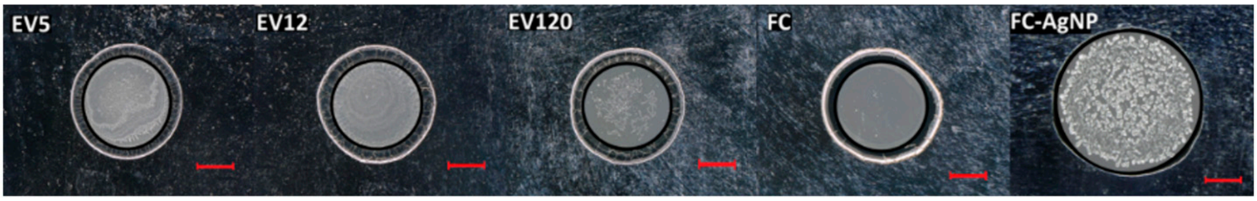

SI Figure 4: Microscopic images of EV-enriched and fc control fractions subjected to drop-coating deposition and dried on a CaF_2 substrate. Dried samples formed films with so-called "coffee-ring" zone with highest biomolecule concentration at the margin. The diameter of the film with AgNP-KCl was larger due to higher sample volume and showed salt crystals in the center (each scale bar: 1 mm).

References

1. Esmonde-White, K.A.; Esmonde-White, F.W.L.; Morris, M.D.; Roessler, B.J. Characterization of biofluids prepared by sessile drop formation. *Analyst* **2014**, *139*, 2734-2741, doi:10.1039/c3an02175k.

2. Krafft, C.; Wilhelm, K.; Eremin, A.; Nestel, S.; von Bubnoff, N.; Schultze-Seemann, W.; Popp, J.; Nazarenko, I. A specific spectral signature of serum and plasma-derived extracellular vesicles for cancer screening. *Nanomedicine: Nanotechnology, Biology and Medicine* **2017**, *13*, 835-841.
